# Textile-Based Mechanical Sensors: A Review

**DOI:** 10.3390/ma14206073

**Published:** 2021-10-14

**Authors:** Zaiwei Zhou, Nuo Chen, Hongchuan Zhong, Wanli Zhang, Yue Zhang, Xiangyu Yin, Bingwei He

**Affiliations:** 1 College of Mechanical Engineering and Automation, Fuzhou University, Fuzhou 350108, China; zzw2400@163.com (Z.Z.); N190220082@fzu.edu.cn (H.Z.); 13215041192@163.com (W.Z.); 2 Department of Mechanical and Energy Engineering, Southern University of Science and Technology, Shenzhen 518055, China; 11810222@mail.sustech.edu.cn; 3 Fujian Engineering Research Center of Joint Intelligent Medical Engineering, Fuzhou 350108, China; 4 College of Chemical Engineering, Fuzhou University, Fuzhou 350108, China

**Keywords:** textile-based mechanical sensors, mechanism, preparation, advantages, applications

## Abstract

Innovations related to textiles-based sensors have drawn great interest due to their outstanding merits of flexibility, comfort, low cost, and wearability. Textile-based sensors are often tied to certain parts of the human body to collect mechanical, physical, and chemical stimuli to identify and record human health and exercise. Until now, much research and review work has been carried out to summarize and promote the development of textile-based sensors. As a feature, we focus on textile-based mechanical sensors (TMSs), especially on their advantages and the way they achieve performance optimizations in this review. We first adopt a novel approach to introduce different kinds of TMSs by combining sensing mechanisms, textile structure, and novel fabricating strategies for implementing TMSs and focusing on critical performance criteria such as sensitivity, response range, response time, and stability. Next, we summarize their great advantages over other flexible sensors, and their potential applications in health monitoring, motion recognition, and human-machine interaction. Finally, we present the challenges and prospects to provide meaningful guidelines and directions for future research. The TMSs play an important role in promoting the development of the emerging Internet of Things, which can make health monitoring and everyday objects connect more smartly, conveniently, and comfortably efficiently in a wearable way in the coming years.

## 1. Introduction

As measuring devices, mechanical sensors can obtain mechanical stimulus information and convert it into electrical signals to achieve functions such as stimulus acquisition, transmission, and storage. When mechanical stimuli, such as pressure, strain, motion, etc., is applied, the mechanical sensor can convert it into electrical signals such as resistance, voltage, and capacitance, and based on the mechanical stimulus-electrical signal regression curve, we can infer the magnitude of the mechanical stimulus. In accordance with different mechanical stimuli, mechanical sensors can be substantially divided into strain sensors, pressure sensors, position sensors, velocity sensors, tactile sensors, etc. Traditional mechanical sensors made of metal, semiconductor, or ceramic have stable performance and mature preparation technology, which have played a major role in traditional industries. However, their inherent shortcomings, such as rigidity, large size and weight, and small deformability, indicate that they are not applicable to the fields of electronic skins, human physiological signals, intelligent robots, etc. [1]. Flexible materials usually feature softness, easy deformation, and light weight [2,3]. Some novel flexible materials exhibit unique properties such as self-healing, hydrophobicity, biocompatibility, and biodegradability [4,5,6], which make them more competitive when compared with rigid mechanical sensors [7,8,9,10]. Textile materials are considered as promising new versions of silicon wafers in wearable electronics, not only because they have the properties of most flexible materials, but also because they hold the advantages of low cost, good conformality, comfort, and wearability [11]. Therefore, textile materials have shown great application potential in wearable electronics, human-machine interaction, smart fabrics, etc. [12,13,14].

Some applications of textile-based mechanical sensors (TMSs), such as monitoring heartbeat, pulse, and other health signals [15,16,17], constructing robot human-like tactile perception function [18,19,20], and fabricating human-movement monitor smart clothing [21,22,23], are already research hotspots for wearable electronics. In these applications, epidermic sensors are also strong competitors. Recent studies suggest that ultrathin multimodal devices based on piezoelectric or triboelectric polymers may be attractive candidates for the development of imperceptible mechanical sensors in direct contact with the human body [24]. Unlike TMSs, the imperceptible epidermic sensors fit directly on the skin due to their thinness, small size, and even transparency, whereas TMSs are woven into clothing to be hidden. Similarly, tattoo sensors, which can be sub-micron thick and monitor the human body information without affecting the senses and aesthetics, are attracting the interest of researchers [25]. Compared to other types of mechanical sensors, TMSs have natural advantages in monitoring large areas, large deformation mechanical signals, and biocompatibility due to their weaving method and textile substrates.

However, the research on TMSs is still in the laboratory stage, and there are many scientific and technical problems to be solved, such as the inability to balance good electrical conductivity and air permeability, poor durability, and improved integration of active materials and fabrics, etc. Although some reviews have been reported on the principles, materials, preparation methods, and application areas of textile-based sensors [26,27,28,29], there are few reviews that systematically introduce TMSs. Here, we adopt a novel approach to introduce different kinds of TMSs by combining sensing mechanisms, textile structure, and novel fabricating strategies. In particular, we highlight the unique pros and cons of the TMSs, and summarize methods to improve existing deficiencies. To summarize the recent progress, we focus on the latest results and applications of TMSs, and for these, we present the prospects and challenges of TMSs.

In this review, we systematically introduce features and latest achievements in TMSs. Four types of sensing mechanisms of resistance, capacitance, piezoelectricity, and triboelectricity of TMSs as well as the configuration features of each type of device and their corresponding preparation and integration methods are introduced. The commonly used substrate materials, sensor materials, and preparation methods of conductive fiber, yarn, or fabric are also summarized. Next, the advantages and sensing performances of TMSs are analyzed, and the designs related to improving the performance and advantages of TMSs are discussed. The advanced applications based on TMSs are then described in detail. Finally, the challenges and development directions of the device are further discussed.

## 2. Sensing Mechanisms, Materials, and Preparations

TMSs can introduce electrical conductivity in three structural forms: fiber, yarn, and fabric [25]. Among them, conductive fibers can be inherently conductive metal wires of silver, copper, and stainless steel, or they can be made by blending conductive materials, such as polyaniline (PANI), polypyrrole (PPy), and graphite, carbon nanotubes (CNTs) into natural (e.g., cotton, silk, and bamboo) and synthetic (e.g., nylon, vinylon, and polypropylene) fibers, or by directly coating or plating these conductive materials onto polymer (polypropylene (PP), polyethylene terephthalate (PET), polyimide (PI), etc.) fibers [30]. The obtained conductive fibers can be twisted to make conductive yarns, and by subsequent weaving and knotting can be made into conductive fabrics. In addition, conductive fabrics can also be made by directly dip-coating, spin coating, or printing conducting inks onto normal fabrics. TMSs in three structural forms of fiber, yarn, and fabric can be integrated into textiles or woven into clothing. Fibers or yarns are the first choice for TMSs as they can be easily converted into fabrics or combined with conventional fabrics by textile techniques (e.g., weaving or knitting). TMSs are mainly categorized as resistive, capacitive, piezoelectric, and triboelectric [31] according to their sensing mechanism. The different sensing mechanisms and their implementation methods are described in detail below.

### 2.1. Resistive Sensor

Resistive TMSs convert mechanical stimuli, such as displacement or force to a resistance change, using piezoresistive materials. As the resistance of a conductive material is defined as R = ρL/S, when a mechanical stimulus causes changes of the piezoresistive materials in resistivity (ρ), length (L), and/or cross-sectional area (S), it will bring out a resistance change. The sensing response of resistive TMSs depends on the interaction of these main factors: (1) intrinsic changes in the resistance of sensing elements in response to mechanical stimuli; (2) geometric variation of assembled devices; and (3) changes in the conduction network. Based on a regression curve, the mechanical stimuli and their degrees can be determined. Because resistive TMSs often have the advantages of high sensitivity, wide detection range, high precision, and simple measurement circuits, they have received the most extensive attention and study [32]. However, large signal drift, poor durability, and obvious hysteresis are the key issues that restrict their practical application [33].

**Figure 1:**
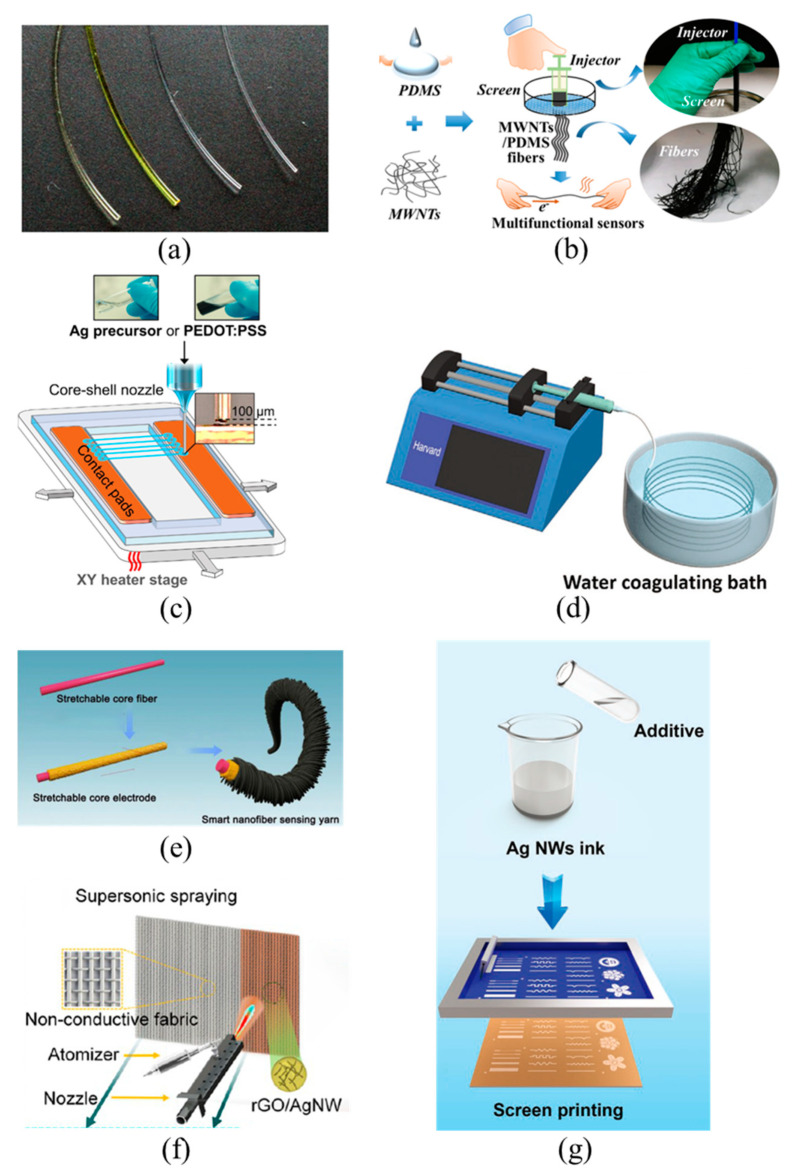
Illustration of the manufacturing process of various resistive textile-based mechanical sensors (TMSs). PDMS means polydimethylsiloxane, MWNTs means multi-walled carbon nanotubes, PEDOT:PSS means poly(3,4-ethylenedioxythiophene)/poly(styrenesulfonate), rGO means reduced graphene oxide and AgNWs means silver nanowires. Fiber-based sensors prepared by (**a**) coating, reproduced with permission from [35]; (**b**) extrusion, reproduced with permission from [36]; (**c**) printing, reproduced with permission from [37]; and (**d**) wet spinning, reproduced with permission from [38]; (**e**) Yarn-based sensors prepared by electrostatic spinning, reproduced with permission from [39]; Fabric-based sensors prepared by (**f**) spraying, reproduced with permission from [41]; and (**g**) screen printing, reproduced with permission from ref. [42].

Resistive TMSs are typically composed of a soft substrate and a sensing material [34]. The soft substrate needs have properties such as a certain elasticity, good flexibility, and long-term stability. These properties can provide a carrier for sensing materials, and endow the sensing materials and subsequent textiles with piezoresistive properties. They can also reduce the stress concentration of TMSs when subjected to mechanical stimuli. Commonly used substrate materials for TMSs include silk, cotton, polydimethylsiloxane (PDMS), polyurethane (PU), etc. Correspondingly, commonly used sensing materials have carbon materials, metal materials, conductive polymers, etc. The sensing materials need to be both conductive and mechanically robust, which are typically prepared by coating, depositing, winding, or electroplating functional conductive layer on fibers, yarns, or fabrics, and they can also be prepared by wet spinning or 3D printing processes. Functional fibers and yarns can be attached to complex surfaces or woven into fabrics, which makes the sensors adaptable to different application scenarios by changing their shape and can be prepared in a sustainable and large-scale way. Neves et al. [35] produced conductive fibers by coating graphene onto polymer fibers that could be bent, stretched, compressed, twisted, and deformed into complex shapes while still maintaining good performance and reliability (Figure 1a). These graphene-based conductive fibers can be utilized as a platform for constructing integrated electronics directly in textiles. The stretch property of the conductive coating layer is often significantly lower than that of the textile substrate, which often leads to the formation of cracks in large deformations, resulting in reduced sensing stability. Additionally, as the textile surface is coated with a relatively rigid layer of conductive material, the feel and comfort of the fabric will be different. Composite conductive fiber—fiber adding conductive materials—can maintain the characteristics of the main fiber, such as the feeling and wear resistance. The structure of composite fibers is more integrated than that of coated textiles, effectively improving the problem of micro-cracks on the surface of coated fibers. Li et al. [36] used a syringe to extrude a mixture of conductive multiwalled carbon nanotubes (MWCNTs) and PDMS through a mesh with micron-sized holes to fabricate functional fibers (Figure 1b). The fibers as part of a wearable sensor were then integrated into a smart glove to recognize finger dexterity, gestures, and temperature signals. This preparation method is simple and convenient, but it is not suitable for large scale preparation of functional fibers. Wang et al. [37] used a concentric nozzle to rapidly and precisely print nanofibers with a bilayer structure (Figure 1c). The inner layer of the nanofiber acts as a sensing layer composed of metal or conductive polymer materials, whereas the outer layer acts as a protective and supportive layer that consists of long-chain polymer materials. The smart mask made from the nanofibers by a one-step progress can be used to detect whether the mask is worn properly and whether breathing is abnormal. Additionally, traditional textile manufacturing technologies of wet spinning and electrostatic spinning are also applicable to fabricate functional fibers. Sheng et al. [38] prepared porous fiber-based strain sensors by the wet-spinning method, wherein thermoplastic polyurethane was used as elastomer and carbon nanotubes (CNTs) and graphene as conductive fillers (Figure 1d). Before wet-spinning, dispersants and binders were introduced to improve the interaction between the elastomer and the conductive fillers to achieve the purpose of effectively withstanding external forces. Qi et al. [39] used a simple electrostatic spinning technique to prepare nanofiber sensing yarn, which was composed of a fibrous core electrode wrapped and wound by piezoresistive elastic nanofibers (Figure 1e). The yarn showed a fine layered structure, and could be woven into fabrics to achieve multi-mode sensing of various mechanical stimuli.

Fabric-based mechanical sensors can also be designed and prepared by the methods of coating, deposition, inkjet printing, screen printing, etc. Among them, directly coating sensing material onto common fabrics is the simplest and easiest method to achieve large-scale TMSs preparation. However, this method will bring about a poor bond between the sensing material and the flexible fabrics, so that the stability and durability of the prepared TMSs cannot be guaranteed. Thus, how to improve the adhesion between the two materials has become the first problem to be solved in the preparation of high-performance TMSs, wherein functionalized molecular grafting sensing materials is one of the preferred methods. Liu et al. [40] coated fluorinated MXene nanosheets onto 15 different fabrics, because the surface of the MXene is rich in a large amount of functional groups that interact with the fabric surface to improve adhesion between the two. It has been experimentally proven that the MXene formed a strongest bond with pure cotton and will not come off even after washing and ultrasonic processing. In addition, the adhesion can be enhanced by improving the preparation process of the TMSs. A multifunctional mechanical-sensitive fabric is prepared via ultrasonically spraying reduced graphene oxide (rGO) and silver nanowires (AgNWs) onto synthetic and 100% natural cotton fabrics (Figure 1f). The obtained fabrics show a good durability and can be washed repeatedly without performance degradation [41]. Luo et al. [42] used simple and efficient screen printing to transfer high-performance AgNW inks onto stretchable fabrics (Figure 1g), which presented excellent tensile properties and sensing performance. Conveniently, sensing materials with different patterns can be printed by simply changing the screen with different shapes, and fabric-based mechanical sensors prepared by printing processes can be designed into desired patterns to improve the sensing range, sensitivity, and other properties of resistive sensors. In summary, the coating and screen-printing method is easy to implement TMSs with superior sensitivity and a relatively large sensing range. However, the low linear correlation and cyclic stability of the sensing layer greatly limit its practical application.

### 2.2. Capacitive Sensor

Capacitive TMSs are realized based on the capacitance changes of the sensing devices induced by external mechanical stimuli. The capacitance of a sensing device is defined as C = ε_r_ε_o_A/d, where ε_r_ is the relative permittivity, ε_o_ is the vacuum permittivity, A is the effective area of the electrode, and d is the pole-plate spacing [43]. Therefore, the change of one or more parameters of the permittivity, spacing, or effective area will cause a change in capacitance of the device, and then the magnitude of the mechanical stimulus that causes the parameter change can be quantified. Capacitive TMSs feature properties of high response repeatability, small signal drift, long term cycle stability, and low energy consumption, but they are susceptible to external field interference, relatively low sensitivity, and limited sensing range [44].

In contrast, the fabrication of capacitive TMSs is more challenging than for resistive ones, because capacitive TMSs usually consist of two electrode layers and a dielectric layer [45], wherein the electrodes require good electrical conductivity; commonly used electrode materials include conductive fabrics, metal wires, carbon materials, etc. Although each electrode needs to be conductive, the capacitive response is irrelevant to the change in resistance of the electrode during exposure to mechanical stimulation. At the same time, the materials used as dielectric layers usually possess a large dielectric constant to reduce leakage current. Commonly used dielectric materials are elastic polymers, fabric gaskets, ionic gels, etc.

**Figure 2:**
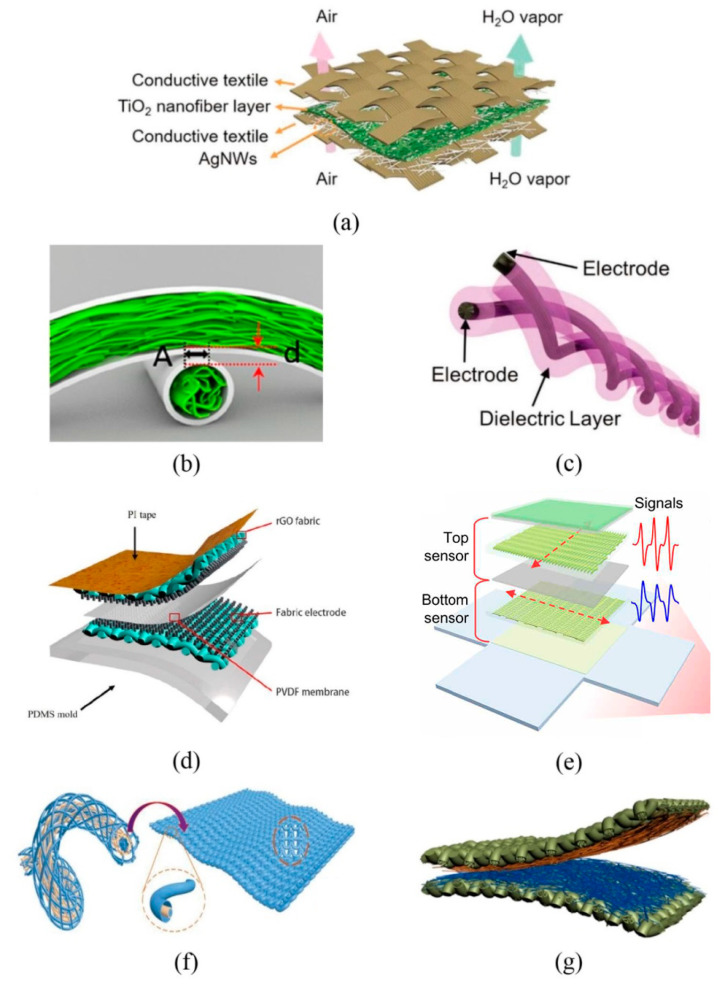
Schematic diagrams of TMSs configurations based on capacitive, piezoelectric, and triboelectric sensing mechanisms. PDMS means polydimethylsiloxane, PI means polyimide, rGO means reduced graphene oxide and PVDF means polyvinylidene fluoride. (**a**) sandwich structure fabric capacitive sensors, reproduced with permission from [47]; (**b**) crossed fiber capacitive sensors, reproduced with permission from [48]; (**c**) helix fiber capacitive sensors, reproduced with permission from [49]; (**d**) sandwich structure piezoelectric sensors, reproduced with permission from [50]; (**e**) double-layer piezoelectric sensors with vertical arrangement, reproduced with permission from [51]; (**f**) coaxial fiber triboelectric sensors, reproduced with permission from [52]; and (**g**) double-layer triboelectric sensors, reproduced with permission from ref. [53].

The preparation method of the capacitive TMSs is similar to that of the resistive ones, but the configurations of the two devices are different. The device configuration of a capacitive TMS can be roughly divided into two types. The first configuration is constructed as a sandwich structure, which consists of two flat electrodes composed of conductive fabrics and a dielectric layer composed of common fiber membranes or ionic gel membranes sandwiched between the electrodes. This type of structure is the most common one, which usually endows the device with a large sensing range. Keum et al. [46] prepared sandwich-structure ion-electron pressure TMSs using silver-plated compound silk fibers as electrodes and high-permittivity ion gel membranes as the dielectric material. The composition and membrane thickness of the ionic gel were designed to maximize the change of the contact area between the conductive fabrics and the ionic gel under external forces, which in turn optimized the sensing performance of the devices. Fu et al. [47] prepared a flexible pressure TMS by using fabrics sprayed with AgNWs as the flexible electrodes and a ceramic nanofiber film fabricated via an electrostatic spinning process as the dielectric layer (Figure 2a). The obtained pressure sensor can be used to detect human health conditions and motion, such as pulse, vocal cord vibration, and body movement, etc. The second type of configuration of capacitive TMSs is equipped with a core-sheath structure fabricated by coating and coaxial spinning. Compared with the capacitive TMSs with a sandwich structure, the TMSs with a core-sheath structure are smaller and easier to embed into clothes. Capacitive TMSs can be obtained by fiber crossing. Guan et al. [48] prepared silver nanowire-bacterial cellulose fibers with porous structures using a wet-spinning process and then coaxially coated the fibers with PDMS to produce functional fibers with a core-sheath structure (Figure 2b). A capacitive multifunctional sensor was fabricated by arranging the functional fibers crosswise to form an interpenetrating network, in which the AgNWs-bacterial cellulose fibers served as electrodes and the PDMS coating acted as the dielectric layer. By detecting changes in capacitance, the sensor could detect both the pressure and the position of objects. Additionally, Zhang et al. [49] prepared a high-performance capacitive strain sensor by twisting two core-spun yarns into a fine double-ply yarn (Figure 2c). The core-spun yarns were fabricated by wrapping silver-coated nylon fibers with cotton fibers, and then they were fixed with polyurethane. The sensor exhibits good linearity and tensile properties and can be blended into wearable fabrics to monitor athletes and patients without compromising lifestyle or comfort.

### 2.3. Piezoelectric Sensor

Piezoelectric TMSs are produced from flexible materials with piezoelectric effects, which work by converting mechanical stimuli into voltage signals [54]. The piezoelectric constant of the piezoelectric material determines the performance of a piezoelectric sensor in converting mechanical energy into electrical energy. Commonly used piezoelectric materials include composites, polymers, ceramics, single crystals, etc. [55].

Piezoelectric TMSs can generate internal voltage when subjected to external pressure, which makes them self-powered while achieving pressure sensing. In addition, such TMSs often present the advantages of fast response time and high sensitivity [56], giving them great prospects in wearable devices. Tan et al. [50] prepared piezoelectric TMSs using the piezoelectric effect of the single-crystalline ZnO nanorods grown on conductive rGO-PET fabric (Figure 2d). The piezoelectric TMS is constructed with three layers consisting of polyvinylidene fluoride (PVDF) membrane, the top and bottom electrode layers of conductive rGO-PET fabrics with self-orientation ZnO nanorods. When subjected to an external force, the piezoelectric configuration deformed, leading to a potential difference between the two electrode layers so the magnitude of external force can be obtained by detecting the voltage change. Hong et al. [51] provided a new solution of designing anisotropic kirigami structures and manufacturing a functional piezoceramic network to monitor joint motions and distinguish between different motion modes, in which the piezoelectric composite is the core sensory element for the sensors, formed by a lead zirconate titanate (PZT) ceramic network with nylon textile with kirigami-structured honeycomb grids and a PDMS matrix (Figure 2e). Piezoelectric sensors show obvious advantages in measurement range, piezoelectric anisotropy, multifunctional measurement, and long-term monitoring, which greatly enhance their practical application range.

### 2.4. Triboelectric Sensor

The frictional initiation effect is a normal phenomenon in daily life. It occurs when a material is subjected to normal contact, sliding, or twisting. The combining of the electrification effect/triboelectric effect and electrostatic induction is the principle of triboelectric TMSs that occurs among a broad range of materials, including synthetic polymers and natural silk [57], wool [58], and cotton [59]. Similar to piezoelectric sensors, triboelectric TMSs can convert mechanical motion into electrical signals, and in turn, by analyzing the obtained signals, dynamic mechanical motions can be interpreted. By correlating the mechanical input with the corresponding parameters, a series of triboelectric TMSs have been fabricated, including pressure sensors, strain sensors, and vibration sensors. Triboelectric TMSs are generally composed of two electrodes with different tribo-polarities; the greater the difference in tribo-polarities between the two electrode materials, the better the electrical performance of the sensors [60]. Commonly used positively charged materials include nylon, cotton, silver, and copper, whereas commonly used negatively charged materials include PDMS, PVDF, polytetrafluoroethylene (PTFE), etc. [61]. The advantages of triboelectric TMSs, such as low cost, simple preparation process, high output voltage, and self-power, will strongly promote the construction of the Internet of Things [62].

Triboelectric TMSs can be divided into two kinds. One kind is a single yarn device with two frictional electrical sequences that are woven into fabrics or textiles. Zhang et al. [52] prepared a coaxial triboelectric yarn by sequentially wrapping PTFE and Ag yarns around axial metallized silver yarn via a winding machine (Figure 2f). The fabricated triboelectric yarn was then woven into wearable, multifunctional textile by needles to harvest the mechanical energy from human body motions. Since the tribo-polarities between PTFE and Ag materials differ greatly, charge transfer was easily achieved in repeated contact–separation processes. The second kind of triboelectric TMSs is obtained by directly weaving two types of yarns or fibers with inherently different tribo-polarities. Fan et al. [63] wove terylene wrapped stainless steel conductive yarns and nylon yarns into an all-textile triboelectric sensing array. Guo et al. [53] fabricated a textile based wearable hybrid triboelectric-piezoelectric TMS composed of silk fibroin nanofibers and PVDF nanofibers that were electrospun onto conductive fabrics as the triboelectric pair. Before fabricating a cloth-shape smart device, the two triboelectric fabrics were attached to separate substrates to realize effective contact and separation (Figure 2g). The hybrid TMS is capable of generating both triboelectricity and piezoelectricity at the same time to realize high power generation that enables its use as a sensor to identify various types of body motion without another power supply.

Each of the different types of TMSs has its own pros and cons. Resistor TMSs are by far the most widely studied and applied due to the simple principle and read-out circuit. Compared with other types, capacitor TMSs have a longer lifetime due to lower heat generation. In addition, they also show applications in non-contact measurements because of the sensing mechanism. Piezoelectric and triboelectric TMSs are difficult to detect static forces due to charge loss. Despite the fact that piezoelectric and triboelectric TMSs are limited by the inability to detect static forces and their applications are restricted, they are still a current research hotspot owing to their huge advantages of self-powering. How to overcome the shortcomings of piezoelectric and triboelectric TMSs that cannot detect static forces is a major challenge. The resistive and capacitive TMSs have wider applications in most scenarios. However, in some harsh environments, they need to replace the power supply in time, which necessitates more labor and material resources. Whereas the piezoelectric and triboelectric TMSs are self-powered, and the signal can be detected in the field for a long time, which greatly saves cost.

## 3. Advantages and Performance

### 3.1. Advantages

**Figure 3:**
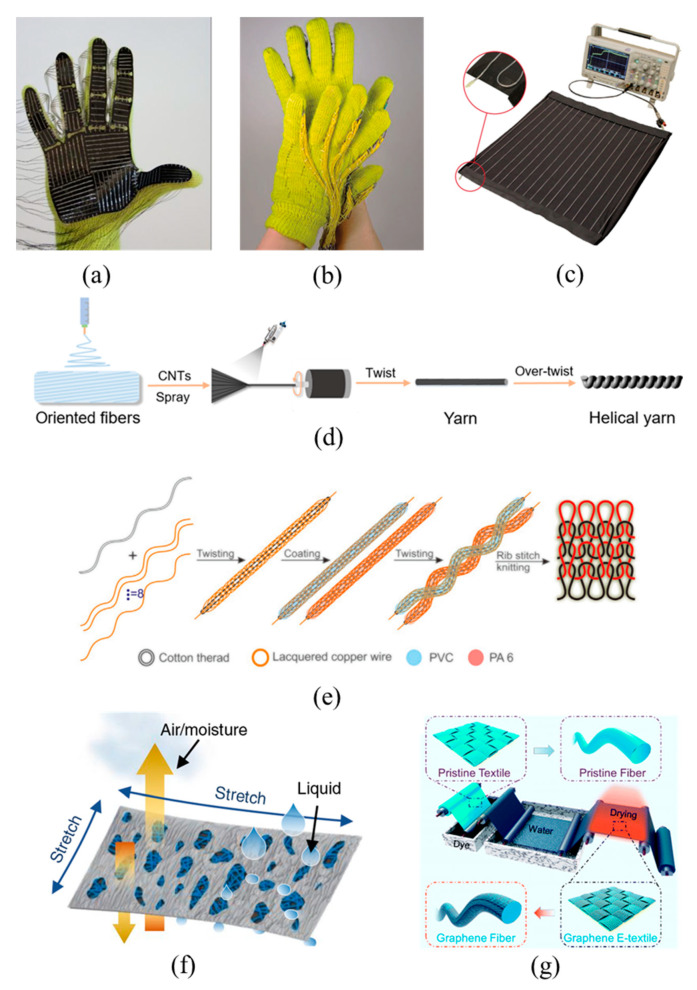
Advantages. CNTs means carbon nanotubes, PVC means poly vinyl chloride and PA6 means Polyamide 6 (**a**) large area array sensors based on piezoresistive film, reproduced with permission from [64]; (**b**) large area array sensors based on fibers, reproduced with permission from [65]; (**c**) large area detection mechanical stimulation using electrical time domain reflectometer, reproduced with permission from [66]; (**d**) large deformation sensors based on spiral structure, reproduced with permission from [68]; (**e**) large deformation sensors based on ribbed weave, reproduced with permission from [69]; (**f**) fabric sensors breathability based on pre-stretching, reproduced with permission from [72]; and (**g**) fabric sensors breathability based on coating shrinkage, reproduced with permission from ref. [73].

To identify and quantify mechanical stimuli in space, the method of arranging sensing units in membranes or blocks on a flexible substrate is usually adopted. This method is indeed very simple and practical in the application situation where the precision of mechanical stimuli is not high. However, the large area of each sensing unit, as well as the messy electrode port (generally, n sensing units need n + 1 electrode ports) and complex circuit layout result in the sensing units not being sufficiently close, hence the integrated devices often show a low spatial resolution in detecting mechanical stimuli. In contrast, TMSs offer great advantages over other flexible mechanical sensors in terms of large area manufacturing. The TMSs, using fine fibers, yarns, or fabrics as a distributed sensing network, greatly reduce the number of electrode ports (generally, m × n sensing points can be arranged with m + n electrodes) and achieve a high-resolution spatial recognition of mechanical stimuli. Sundaram et al. [64] assembled a sensor array on a knitted glove to detect tactile information (Figure 3a). The sensing array consists of a piezoresistive membrane connected by a network of conductive wire electrodes. This approach allows the detection of mechanical stimuli with very high spatial resolution. In addition to the use of piezoresistive membranes, sensing arrays can also be prepared directly using piezoresistive fibers. Luo et al. [65] proposed a strategy to prepare fibers by coating conductive stainless steel wires with piezoresistive nanocomposites using an automated coating technique (Figure 3b). Taking advantage of this strategy, the fibers can be fabricated into a sensing unit by a simple vertical overlapping progress, and can be subsequently woven into large-scale sensing textiles with arbitrary 3D shapes for spatially accurate detection of mechanical stimuli. In addition to preparing an array sensor, fibers are also able to detect mechanical stimuli in space by using an electrical time-domain reflectometer to send pulses to a separate transmission line, and the amplitude of the step indicates the magnitude of the pressure and the occurrence time of the step indicates the distance. For example, Leber et al. [66] prepared an elastic fiber that integrated dozens of liquid metal conductors with a uniform, complex cross-sectional structure. The fiber was arranged in a snake shape onto stretchable fabric, which was connected to an electrical time-domain reflectometer via a single contact (Figure 3c). An electrical time-domain reflectometer could detect the location and magnitude of mechanical stimuli by transmitting high-frequency pulses to a transmission line and then reflecting them at discontinuities. This approach makes the structure of the sensing fabric simpler, with only one electrode port. However, it is difficult to integrate the sensing fabric into a wearable device due to the need of an electrical time-domain reflectometer.

During practical applications, mechanical sensors are subject to various mechanical stimuli such as stretching, squeezing, bending, etc., which require a good deformability of the electrodes and sensing elements in mechanical sensors, a stable sensing function in a large deformation state, and a structural and electrical performance reversibility after the release of deformation [67]. TMSs have unique advantages in terms of deformability, which is generally obtained by optimal structural and material design. Gao et al. [68] uniformly sprayed CNTs dispersion onto PU nanofiber substrate and then obtained CNTs/PU fibers with helical structure by twisting to an over-twisting state. The CNTs/PU fibers could reach 900% deformation range (Figure 3d), because the helical structure has the advantage in tensile strain and strength compared to fiber film and fiber bundle structures, which underwent unscrewing during the stretching process. Importantly, the spiral fibers could recover their original structure after stress release due to the PU elastic substrate, and the cracks in the conductive network of CNTs were repaired. Therefore, sensors are highly reversible in terms of structural and electrical properties. In addition to improving the deformation performance by designing the fiber structure, the deformation capacity of the fabric can also be improved by studying the weaving process and weaving structure. Rezaei et al. [69] twisted cotton threads with eight copper wires and then coated them with reactive triboelectric materials poly vinyl chloride (PVC) and polyamide 6 (PA 6), respectively. The two types of fibers were twisted and prepared into a double twisted thread as a triboelectric sensor. By studying the weaving process, the ribbed weave was chosen to endow the fabric with excellent tensile properties and flexibility (Figure 3e). Another key to improving the deformability of TMSs is material optimization through the development of materials with inherent deformation characteristics or the integration of deformable materials. Commonly used materials are hydrogels, PDMS, etc. Ye et al. [70] prepared a conductive hydrogel by integrating polymer, silk protein, and carbon materials, which had a strain range of 600% and showed a rapid recovery ability during large deformations.

For wearable applications, TMSs are required to have the feel of textiles and be comfortable to wear. When sensors are worn or fitted to the body, they should not cause any discomfort to the body, and therefore require good biocompatibility, skin friendliness, and comfort. Mechanical sensors that use textile materials as substrates usually possess these characteristics [71]. TMSs can be sewn on the garment manually by attaching them to an elastic backing or by using a specially designed support frame. Further, TMSs are woven directly into textiles that can be worn directly without support frames, backing, or other clothing items, allowing wearable TMSs to take a step forward in comfort and appeal [65]. In addition, breathability is also a major focus of the sensors. Fabrics woven with fiber-based TMSs usually present good air breathability due to the voids between the fibers, whereas TMSs with a coated sensing layer usually need to be designed as a porous or mesh structure to provide breathability. For instance, Ma et al. [72] fabricated a breathable liquid metal-fiber layer by coating liquid metal on an elastic fiber layer (Figure 3f). During the fabricating process, pre-stretching was applied to make the liquid metal suspended between the elastic fibers self-organize into a transverse mesh and a vertically curved structure, thus providing the liquid metal-fiber layer with good breathability. The combination of hydrophobicity and washability, which are not available in other types of flexible mechanical sensors, makes TMSs more attractive. The combined characteristics can be possessed by material design without affecting breathability. Hu et al. [73] obtained graphene coated e-textiles through dipping, water treatment, and a subsequent drying step (Figure 3g). After dipping, the fabric is immersed in a water bath where the rapid separation of solvent and water causes the coating to shrink, allowing the fabric structure to remain breathable. At the same time methyltrichlorosilan reacts with water to form a methyl-trihydroxysilane precursor, which undergoes condensation to form a highly hydrophobic and sticky polymethylsiloxane structure in a high temperature drying step. Even when textiles are immersed in detergent under ultrasonic washing conditions, the coating remains stable and does not peel off, demonstrating excellent washability. The microstructure design also allows the fabric to be hydrophobic. Inspired by the “papillae structure” on the surface of lotus leaf, Song et al. [74] successfully prepared a waterproof multimode sensor by constructing two-dimensional MXene nanosheets and zero-dimensional silicon nanoparticles on a cotton fiber substrate. The conductivity can be maintained even under wet and corrosive conditions.

Compared with epidermic sensors and tattoo sensors, which are also hotspots in wearable device research, TMSs have the inherent advantage of monitoring large-area, large-deformation mechanical signals and biocompatibility because of their weaving method and textile substrate. Even though TMSs may not be as thin and transparent as other sensors, TMSs can be woven into clothing to hide themselves quite well. TMSs integrated in clothing have more comfort to monitor mechanical signals; additionally, although the skin-conformality is not as good as epidermic sensors and tattoo sensors, TMSs can also be attached to the skin directly for more accurate signals by traditional adhesives. The weaker adhesion between the TMSs and the body causes loss of contact of TMSs from the human skin, resulting in larger noise and inaccurate mechanical signals. To solve this problem, it is necessary for new technologies to replace traditional adhesives to ensure conformal contact between TMSs and human skin.

### 3.2. Performance

So far, a lot of work has been carried out on the developing and research of TMSs to improve the sensing performance of the devices, such as sensitivity, response range, response time, stability, etc., because these properties determine the practical application capabilities of the sensors [75]. Sensing performance can be improved by introducing special geometric structures, such as microarrays [76,77], microcracks [78], micropatterns [79,80], pleated structures [81,82], porous structures [83,84], spiral structures [85,86], etc. The innovation of materials is also a major key point to improve the sensing performance. For example, new materials such as graphene [87,88] and MXene [89,90] have good electrical conductivity and mechanical properties and are widely used in sensors.

**Figure 4:**
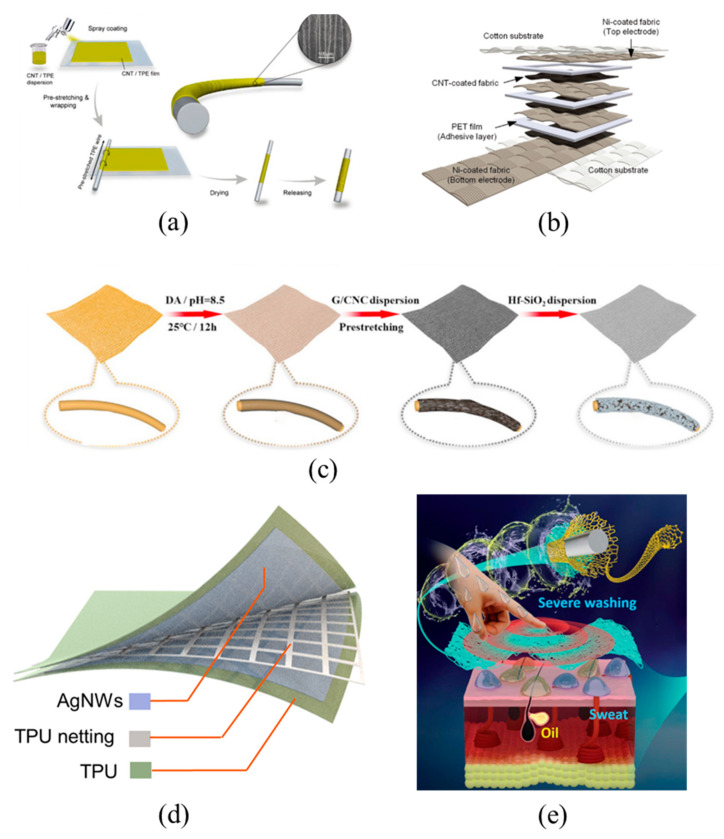
Performance. TPE means thermoplastic elastomer, CNT means carbon nanotube. PET means polyethylene terephthalate, G/CNC means conductive cellulose nanocrystal/graphene, AgNWs means silver nanowires and TPU means thermoplastic polyurethanes. (**a**) high sensitivity based on folded structure, reproduced with permission from [92]; (**b**) large response range based on hierarchical and multilayer structure, reproduced with permission from [91]; (**c**) weighing sensitivity and response range based on microcrack density, reproduced with permission from [93]; (**d**) short response time based on micropatterned dielectric.layer, reproduced with permission from [94]; (**e**) excellent stability based on layered protection structure, reproduced with permission from ref. [96].

Sensitivity is a key factor in evaluating the performance of various sensors. Therefore, many scholars focus on pursuing high sensitivity. The formula for sensitivity is S = (∆X/X_0_)/Y, where S represents the sensitivity, X_0_ represents the initial value of the electrical signal, resistance, capacitance, voltage, etc., ∆X represents the amount of change in the electrical signal, and Y represents the mechanical stimulus applied to the sensor, such as pressure, strain, etc. The usual methods to improve sensitivity include the introduction of microstructures [78], the use of new sensing materials [80], and the employment of multilayer structures [91]. Li et al. [92] prepared a folded core-sheath structure fiber strain sensor by pre-stretching and releasing ultra-light MWCNTs/themoplasticelastomer (TPE) composite film wrapped TPE fiber core. After releasing the pre-stretched TPE fiber core, a periodic bending structure was formed along the fiber axial direction (Figure 4a), which combined with the super-elasticity of the TPE core and provided this fiber high strain sensitivity.

The sensing range refers to the detection range of sensors working reliably, which should cover the full range of the desired application. The sensing range of a sensor determines its application field. Take gripping as an example: the finger joints bend by up to 30% [34]. Therefore, textile strain sensors should be fully functional in the strain range of 0 to 30% to monitor these relative finger movements. Maintaining a high sensitivity over a wide sensing range is one of the properties pursued by scientists. Using multi-layer structures and increasing the contact area are the main methods to improve detection range at present. Pyo et al. [91] developed a resistive tactile sensor by alternately stacking CNTs and Ni fabrics (Figure 4b). The graded structure of the fabric provides a large surface area and microscopic roughness, thereby significantly increasing contact area in response to pressure. The design of multi-layer structures can further increase the contact area and effectively distribute stress to each layer, consequently dramatically increasing the pressure detection range as well as the device’s sensitivity. The sensor shows a sensitivity of 26.13 kPa^−1^ over a wide pressure range of 0.2–982 kPa. Liu et al. [93] successfully fabricated a microcracked nonwoven strain sensor with a wide operating range and a high sensitivity (Figure 4c). Taking advantage of the difference in modulus between the electrically conductive cellulose nanocrystal/graphene coating and the nonwoven fabric, microcrack structures were constructed by a simple dip-coating and pre-strain technique, which determined the sensor’s detecting range and sensitivity, making it possible to prepare sensors with designed sensing capability by adjusting the density of the microcrack structure.

Response time of a mechanical sensor is defined as the time to achieve a steady-state response upon mechanical stimulation, and it is usually defined as 90% time to reach stability. The response time of a sensor is especially important in applications that require real-time data processing: the shorter the response time, the better the real-time performance of the results. The response time of TMSs is typically in the millisecond range level, which can of course be effectively reduced by structural design and material optimization. Yu et al. [94] prepared an ultrathin all-fabric capacitive sensor with two AgNWs electrodes and a breathable micropatterned nanofiber dielectric layer sandwiched between them (Figure 4d). Due to the unique structure of the micropatterned nanofiber dielectric layer, the sensor shows a response time of 27.3 ms. Xu et al. [95] prepared fabric sensors using laser engraved silver-plated fabric as electrodes and graphite flake modified nonwoven fabric as the sensing material. Due to the unique structure of the electrodes and the random rough surface of the sensing material, the sensor has a fast response time of 4 ms.

Stability is a key factor to ensure that a sensor can effectively acquire mechanical stimuli and respond accordingly. The stability of TMSs includes mechanical stability, chemical stability, and cyclic stability, etc., which can also be improved by optimizing structure and materials. Mechanical and chemical stability refers to the ability of the sensor to resist wear, corrosion, and other external environmental factors. To address the vulnerability of textile-based sensors to external mechanical, chemical, and environmental interference, especially human sweat, grease, and wear and tear, Zhang et al. [96] combined carbon nanotube networks, polymer layers, and textile substrates to form self-protected and reproducible e-textiles with a layered structure (Figure 4e). The sensor exhibits excellent characteristics of superhydrophobicity, wear resistance, and mechanical and chemical stability, and its response resistance does not change significantly during 3000 compression cycles. Cycling stability is the ability of the sensing response to return to its original value after unloading. Resistive TMSs usually rely on large deformation to change the resistance to play the role of sensing. Due to irreversible deformation, they are less cyclically stable compared to other types of sensors. Thus, improved cycling stability can also be done by changing the sensing mechanism. Wu et al. [97] transferred water-soluble poly(vinyl alcohol) template-assisted silver nanofibers onto a fabric surface to serve as sensor electrodes, and used a highly elastic three-dimensional penetration fabric as a dielectric layer. The integrated capacitive TMS were prepared with good dimensional stability and excellent cycling stability (≥20,000).

In addition to some of the properties discussed in detail above, the reply ability [98], crosstalk problems [99], linearity [100], and anti-interference [101] of TMSs are also key factors that determine the overall performance and practical applicability of the sensor. For example, it is highly desirable that the sensing response (resistance, etc.) is linearly related to the strain, as this will allow easy prediction of the strain from the sensing response. Sometimes, the required sensing performance is not consistent for different application scenarios, which requires us to design and prepare TMSs that meet the requirements according to the actual situation. However, high-performance TMSs are still a research hotspot. Therefore, the design of innovative textile materials and structures is necessary to achieve TMSs with high sensitivity and other high performance. From a production point of view, the manufacture of TMSs should be easily scalable and economically feasible, which are important factors that limit the large-scale adoption of TMSs.

## 4. Applications

TMSs have great advantages of easy large-scale preparation, flexibility, and biocompatibility. Combined with integrated circuits, they can obtain real-time data for machine learning and artificial intelligence [102], and thus are receiving increasing attention from academia and industry. The assembly of TMSs with units such as power supplies, signal processing components, communications units, and data management software is also a major difficulty. Although TMSs have not yet been marketed on a large scale like most other flexible electronic devices, a great number of studies have shown that TMSs have promising application prospects in wearable electronics, smart fabrics, robotics, and other fields. It is foreseeable that such devices will change people’s lives and improve people’s quality of life in the near future.

### 4.1. Health Monitoring

Health issues are the most important concern for human beings. Traditional medical services usually require professional physicians and large testing and rehabilitation equipment, which requires patients to go to hospitals for testing and treatment on each occasion. Additionally, it increases the burden of patients and physicians and reduces the timeliness of disease detection and treatment. TMSs are very suitable for being applied in the field of health monitoring, because doctors get health data even without patients leaving home combined with Internet of Things (IOT) technology and TMSs [103]. Utilizing the inherent flexibility and comfort of fabric-based sensors, TMSs can not only be used to detect the wearer’s blood pressure, heart rate, and certain diseases, but also can be used to monitor the wearer’s disease progression or motor symptoms for a long time, will provide an alert of possible health threats to patients or an objective basis for doctors to guide the wearer in medication or rehabilitation training, and will even greatly influence the reform of the medical industry and home care industry. In personal health care monitoring, it is important to identify the location and number of sensors. Common body monitoring areas include the soles of the feet, hands, legs, chest, neck, pulse, etc.

**Figure 5:**
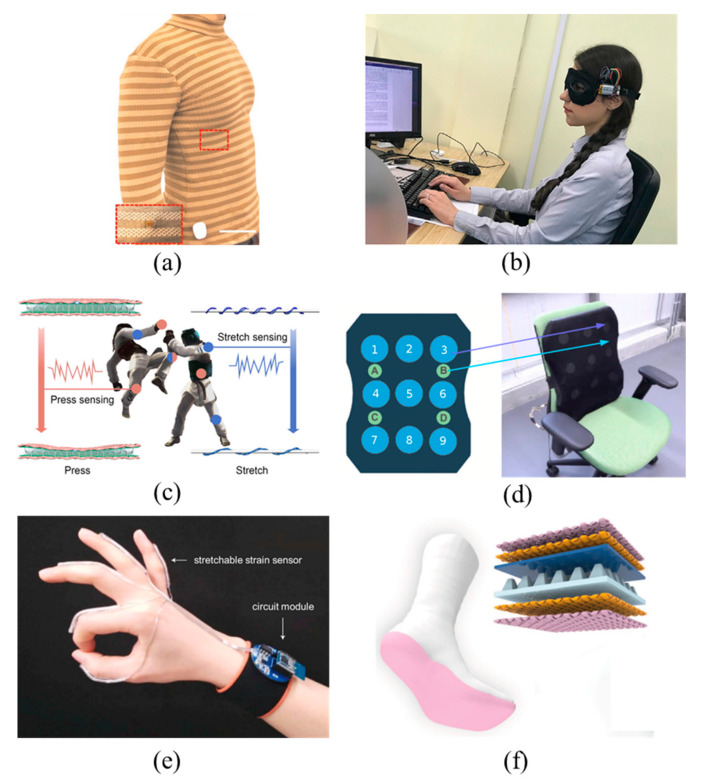
Applications: (**a**) smart clothing applied to health monitoring, reproduced with permission from [104]; (**b**) smart eye mask applied to health monitoring, reproduced with permission from [105]; (**c**) smart clothing applied to motion analysis, reproduced with permission from [106]; (**d**) smart cushion applied to sitting analysis, reproduced with permission from [107]; (**e**) smart glove applied to sign language recognition, reproduced with permission from [108]; and (**f**) smart socks applied to human-machine interaction, reproduced with permission from ref. [109].

Wicaksono et al. [104] reported a large-scale e-textile-based smart clothing (Figure 5a), which is able to perform multi-modal physiological (temperature, heart rate, and respiration) monitoring. Moreover, customized clothing with various forms, sizes, and functions could be made using standard, adjustable, and high-yield textile manufacturing processes and garment patterning techniques. Similar to corsets, the soft and stretchable features of customized smart clothing allow for close contact between the electronic device and human skin, providing physical comfort and improving the anti-interference of sensor. These make it suitable not only in hospitals and laboratories, but also in home care for mobile, comfortable, and continuous physical activity monitoring, which has huge potential in healthcare, rehabilitation, and scientific training.

Most users are very sensitive to the monitoring tools placed on the face or head, and those tools can cause psychological fluctuations that affect the measurement results. The combination of a fabric sensor and clothing can be designed into a wearable device that is not easy to detect or even unnoticeable, so that when it is used as a health detection device, it increases the comfort of the wearer and the accuracy of the test data. For example, when a reusable hydrogel wet electrode and a full-fabric ionic pressure sensor were integrated into an eye mask, the eye mask could track and monitor eye movements and intraocular pressure in daily use [105] (Figure 5b). The technology is virtually non-irritating and non-invasive to the wearer, coupled with its aesthetics and washing stability. When combined with artificial intelligence algorithms, it can be used to accurately monitor the ophthalmology and heart signals required for sleep quality and mental health research.

### 4.2. Motion Recognition for Analysis

Tracking physical activity and habitual movements and analyzing them to extract useful information can be used to scientifically correct training modes and intensity, monitor sitting posture, guide ergonomic design, recognize sign language, etc. Those have become major applications of TMSs. The realization of motion analysis through TMSs first lies in high-precision detection and quantification of various motion variables, such as position, angle, and pressure, and then converting them into visualized electrical signals in real time. Subsequently comprehensively analyzing and establishing the relationship between these electrical signals and human body posture is the key to realizing the high-precision recognition of human spatial motion characteristics.

Smart clothing is often used to monitor human health or exercise. While conventional rigid sensors typically monitor only small strains, TMSs can withstand larger deformations. To detect athletes’ movement in high-intensity physical exercises similar to taekwondo, the smart sportswear is required to withstand large-scale deformation (strain > 50%) and heavy blows (>100 kPa) while maintaining good performance stability. Based on this, Ma et al. [106] used a composite fabric of core-sheath yarn and spacer fabric to prepare an “all-in-one” electronic fabric with a dual tactile and tension stimulus response (Figure 5c). In general, retractable strain sensors with a core-sheath yarn structure can easily measure large movements, yet with a “double solution phenomenon”, that is, a non-monotonic electrical signal response to tensile deformation. To solve this problem, an insulating PU layer was pre-coated on the core-sheath yarns before twisting. This allows the TMS to accurately monitor the exercise action and intensity, thereby implementing its potential application in taekwondo and high-intensity physical exercise analysis.

Low back pain is the most common work-related physical injury among people and is directly related to working in a twisted and bent position for long periods of time. In addition, long-term incorrect sitting posture is not good for people, especially teenagers, because it can cause bone deformation, hunching, curvature of the spine, myopia, and other health problems. To address this issue, Ishac et al. [107] designed a smart cushion, which is flexible, adaptable, and portable (Figure 5d). The cushion consists primarily of a new conductive-fabric pressure sensing array designed and arranged based on human biomechanics, which was used to sense and classify sitting posture (98.1% in accuracy) and adjust upright posture of the user through vibrotactile feedback.

By collecting and analyzing human movement information, TMSs can not only be used to monitor human movement status, assist in training, and guide ergonomic design, but also assist in communication between people. Sign language is a basic communication method for a considerable portion of the population, but it can only be used by trained individuals. To overcome this limitation, Han et al. [108] developed a wearable system that integrates yarn-based stretchable sensors on five fingers (Figure 5e). With this wearable system, sign language gestures can be converted into analog electrical signals and transmitted wirelessly to a portable electronic device. Real-time speech translation can then be achieved using machine learning algorithms and a graphical user interface with recognition rates >98% and recognition times <1 s. An automated sign language recognition system can obviously strengthen the communication function of sign language, making it easier, smoother, and more effective for deaf-mute people to communicate with the outside world.

### 4.3. Human-Machine Interaction

Human-machine interaction is the study of the interaction between a system and its users. In the past, the realization of human-machine interaction usually required bulky, large, and hard devices. Due to the characteristics of E-textile itself, TMSs, as the core of electronic fabrics or wearable devices, provide a more convenient and comfortable channel than other flexible electronics for human-machine interaction. It is possible to control machines or virtual reality (VR) games using TMSs without affecting human physical activity, which has huge impact on industries such as entertainment and leisure and robotics. More and more work is carried out to study and improve the TMSs performance for highly effective human-machine interaction.

TMSs have great potential for human-machine interaction scenarios such as smart furniture, VR games, and robotic control, etc. Gesture recognition occupies an important position in the field of human-computer interaction technology because of its intuitive and convenient advantages. Shuai et al. [110] prepared stretchable, conductive, and self-healing hydrogel sensing fibers by continuous spinning. When the fiber sensors were separately fixed on five fingers, it could monitor and distinguish the movement of each finger by monitoring the electrical signal changes caused by fingers bring bent, and then judge the gestures such as “OK”, “Victory”, and “claw”. The strain sensing capability of this fiber sensor shows its great potential in human-computer interaction. Gait analysis is also commonly used in human-computer interaction and personal healthcare. As we know, the sole of the human foot has different pressure zones; TMSs can generate a map of the pressure distribution on the bottom of the foot. Zhang et al. [109] simulate a virtual reality (VR) fitness game by mapping gait information collected by smart socks to virtual space (Figure 5f). The smart socks were developed by embedding textile-based triboelectric pressure sensors into commercial socks; combined with deep learning, five different gaits were recognized with an accuracy of 96.67%, ensuring the feasibility of the gait control interfaces and promoting the practical application of flexible electronics in the field of the smart home. TMSs are also finding applications in the entertainment field, such as portable textile keyboards. Chen et al. [111] obtained flexible hierarchical helical yarns via coiling the conductive electrodes around a highly stretchable PU (the first helix layer) and super flexible silicon rubber (the second helix layer), respectively. The yarn can work in a strain range of up to 120% and obtain bioenergy to convert into electrical signals that are used to control household devices without amplifying the signal. Interactive electronic pianos and robotic arms can be precisely controlled using the sensors with little noticeable delay, which makes them promising as portable self-powered wearable electronics.

The application of TMSs boils down to acquiring mechanical signals from the human body or robot, and through the processing of data, status information and control signals can be obtained. As artificial intelligence continues to develop, it may even replace humans in making decisions through the mechanical signals acquired.

## 5. Challenges and Prospects

Ingenious structures, perfect processes, and outstanding materials endow TMSs with excellent performance and more prominent advantages over other flexible sensors. The great advantages of TMSs in terms of cost, large-area array, deformation, conformability, wearability, and comfort make TMSs attract extensive research in academia. However, like most flexible electronics, TMSs suffer from a short lifetime compared to rigid sensors, and many performance issues still need to be addressed. Additionally, their roll-to-roll production technology is immature and most TMSs are fabricated in the laboratory or research stages. Many of the reported TMSs are manufactured using manual techniques rather than mechanical engineering. The large-scale manufacturability and reproducibility of e-textiles are uncertain. The future of TMSs depends on the ability to mass produce seamless embedded electronics that meet everyday needs. There are many challenges in moving TMSs to market commercialization: (1) The complex manufacturing methods and expensive materials of some sensors greatly increase cost and reduced practicality; (2) It is difficult to prepare sensors with uniform performance on a large scale; (3) They lack excellent repeatability and robustness, and are often accompanied by poor reliability and life span; (4) The lack of standards makes it difficult for them to be accepted by the general public and the development of production standards will help reduce manufacturing time and final manufacturing costs; (5) The development of flexible integrated circuits and flexible batteries has limited the practical application of TMSs. Once all these challenges are met, mass production of e-textiles will become a reality, which will be a major milestone for wearable devices. New wearable e-textiles provide an opportunity for the market as well as a challenge for interacting with traditional electronic devices. In addition, most of the current TMSs are still used to detect a single or dual mode of mechanical stimuli, mostly pressure or strain, but the mechanical stimuli associated with the human body are much more complex than these. Therefore, how to collect more mechanical stimulus information, how to decouple different types of signals, and how to quantify and spatially resolve multiple mechanical stimuli are the current difficulties faced by TMSs. In the future, textile-based mechanical sensors will move in the direction of high integration, which not only refers to the high integration of sensor fabrication technology and functions, but also refers to the integration of flexible electronic technology with other technologies such as flexible circuits, machine learning, and drive control. What is more, the smart fabric has the functions of responding to environmental stimuli, sensing and driving, and can even adapt to the environment. To address these challenges, with the joint efforts of scientists and engineers from many different disciplines, we believe that TMSs have a bright future and will contribute to the next generation of health monitoring, motion recognition, and human-computer interaction.

## 6. Conclusions

In this work, we have given a comprehensive review of TMSs. Various types of TMSs including resistive, capacitive, piezoelectric, and triboelectric have been introduced combined with materials, structures, and processes. In particular, the advantages and performance of TMSs and their improvement methods are described in detail. We then summarized the latest applications of TMSs in different fields. Furthermore, challenges and perspectives in TMSs were also presented for the current state of TMSs.

## Data Availability

Data sharing not applicable.
